# The longitudinal association between depression, anxiety symptoms and HIV outcomes, and the modifying effect of alcohol dependence among ART clients with hazardous alcohol use in Vietnam

**DOI:** 10.1002/jia2.25746

**Published:** 2021-06-24

**Authors:** Minh X Nguyen, H. Luz McNaughton Reyes, Brian W Pence, Kate Muessig, Heidi E Hutton, Carl A Latkin, David Dowdy, Geetanjali Chander, Kathryn E Lancaster, Constantine Frangakis, Teerada Sripaipan, Viet Ha Tran, Vivian F Go

**Affiliations:** ^1^ Department of Health Behavior Gillings School of Global Public Health University of North Carolina at Chapel Hill Chapel Hill NC USA; ^2^ Department of Epidemiology Gillings School of Global Public Health University of North Carolina at Chapel Hill Chapel Hill NC USA; ^3^ Department of Psychiatry and Behavioral Sciences Johns Hopkins University School of Medicine Baltimore MD USA; ^4^ Department of Health, Behavior and Society Johns Hopkins Bloomberg School of Public Health Baltimore MD USA; ^5^ Department of Epidemiology Johns Hopkins Bloomberg School of Public Health Baltimore MD USA; ^6^ Department of Medicine Johns Hopkins University Baltimore MD USA; ^7^ Department of Epidemiology College of Public Health Ohio State University Columbus OH USA; ^8^ Department of Biostatistics Johns Hopkins Bloomberg School of Public Health Baltimore MD USA

**Keywords:** mental health, depression, anxiety, HIV, hazardous alcohol use, viral suppression, adherence, Vietnam

## Abstract

**Introduction:**

Mental health disorders may negatively impact HIV outcomes, such as viral suppression (VS) and antiretroviral (ART) adherence among people with HIV (PWH) with hazardous alcohol use. This study evaluates the longitudinal association between depression, anxiety symptoms, VS and complete ART adherence among ART clients with hazardous alcohol use in Vietnam; and examines alcohol dependence as a modifier in this association.

**Methods:**

This was a secondary data analysis of a trial for hazardous drinking ART clients in Thai Nguyen, Vietnam. From March 2016 to May 2018, 440 ART clients with an Alcohol Use Disorders Identification Test‐Concise (AUDIT‐C) score ≥4 for men and ≥3 for women were enrolled. Individuals were randomized to either a combined intervention, a brief intervention or a standard of care. Data on sociodemographics, depression, anxiety symptoms, alcohol use, VS and ART adherence were collected at baseline, three, six, and twelve months. Generalized estimating equation models controlling for intervention exposure were used to estimate time‐lagged associations. Risk ratios were estimated using Poisson regression with robust variance estimation.

**Results:**

The mean age of participants was 40.2. The majority was male (96.8%), had at least some secondary school education (85.0%) and had a history of injection drug use (80.9%). No overall effect of depression and anxiety symptoms on VS was observed. When stratified by time, increased anxiety symptoms at six months were associated with VS at 12 months (adjusted risk ratio (aRR) = 1.09; 95% CI 1.02 to 1.17). An increase in depression or anxiety symptoms was associated with a decreased probability of complete ART adherence (depression symptoms: aRR = 0.95; 95% CI: 0.91 to 0.99; anxiety symptoms: aRR = 0.93; 85% CI: 0.88 to 0.99). The negative effects of anxiety symptoms on ART adherence were stronger among participants with alcohol dependence, compared to those without.

**Conclusions:**

Depression and anxiety symptoms had no overall effect on VS, although they were associated with a lower probability of complete ART adherence. Interventions focusing on mental healthcare for PWH with hazardous alcohol use are needed, and integration of mental healthcare and alcohol reduction should be implemented in HIV primary care settings.

## 1 Introduction

People with HIV (PWH) are disproportionately affected by depression and anxiety disorders [1, 2, 3]. Indeed, PWH are 1.6 to 4 times more likely to be diagnosed with depression and anxiety disorders than HIV‐negative individuals [4, 5, 6, 7, 8, 9]. For example a global systematic review reported a prevalence of depression among PWH ranging from 15% to 44%, depending on the region [10, 11]. The presence of anxiety or depression symptoms among PWH has numerous implications for HIV outcomes. The Transactional Model of Stress and Coping suggests that the experience of stress can have a negative impact on physical health and functional status through direct physiological impacts on health or through indirect effects via maladaptive behaviours, such as non‐adherence to medications [12]. Depression and anxiety symptoms among PWH are associated with poor viral load outcomes [3, 13, 14, 15, 16] and lower odds of achieving antiretroviral therapy (ART) adherence [17, 18, 19]. PWH with depression and anxiety symptoms generally have faster progression to AIDS and higher mortality rates [20, 21, 22, 23, 24].

Hazardous drinking is defined as the quantity and pattern of alcohol consumption that increases adverse health outcomes, while alcohol dependence – a higher level of alcohol use disorder – is defined as a strong desire to consume alcohol, difficulties in controlling its use, and persistent use despite harmful consequences [25, 26]. An estimated 25% to 50% of PWH are hazardous drinkers [27, 28, 29], and about 10.6% of PWH had both depression symptoms and harmful levels of alcohol use [30]. PWH with hazardous alcohol use are even more vulnerable to mental disorders than PWH without drinking issues [31], and they may have unique challenges that make abstinence difficult. For example a study among PWH in Vietnam showed that participants were particularly susceptible to alcohol abstinence stigma, which was also associated with higher levels of alcohol use [32]. Therefore, it is essential to understand how depression and anxiety symptoms affect viral suppression (VS) and ART adherence among PWH with hazardous alcohol use. However, there is a dearth of research on the associations between depression, anxiety and HIV outcomes among this subgroup of PWH.

According to the Syndemics Theory, the co‐existence and synergistic interaction of more than one adverse condition in a patient can produce worse health outcomes than each condition independently [33, 34]. While mental health symptoms are independently associated with poorer HIV outcomes, alcohol use can also accelerate HIV progression through a number of mechanisms. High levels of alcohol use do not only negatively impact ART adherence and response to medication but can also lead to compromised liver function and liver diseases [35, 36, 37, 38, 39, 40, 41, 42]. Therefore, mental health symptoms and high levels of alcohol use can substantially increase the risk of treatment failure among PWH [43]. The interrelationship between these comorbidities and HIV outcomes among PWH remains largely unknown. The understanding of how these comorbidities interact will shed light on the need for a more holistic approach to addressing psychological and substance use comorbidities for PWH.

In Vietnam, alcohol is accessible and affordable, and excessive alcohol consumption is common during social and business gatherings [44, 45]. A study among 1016 PWH in Vietnam found that 30.1% of PWH had hazardous alcohol use [46]. Vietnamese PWH are also commonly affected by mental health disorders such as depression and anxiety [47, 48]. Using data from a randomized controlled trial of two alcohol reduction interventions among PWH with hazardous alcohol use in Vietnam, we aim to [1] evaluate the longitudinal association between mental health symptoms (depression and anxiety symptoms) and two HIV outcomes (VS and complete ART adherence) among ART clients with hazardous alcohol use in Vietnam; and [2] determine whether alcohol dependence modifies the longitudinal association between depression, anxiety symptoms and HIV outcomes (conceptual model shown in Figure S1).

## 2 Methods

### 2.1 Study design and study population

This research is a secondary data analysis of the parent study, *Reducing Hazardous Alcohol Use & HIV Viral Load: A* *Randomized Controlled Trial in Antiretroviral Treatment (ART) Clinics in Vietnam* [REDART; NCT02720237]. REDART is a three‐arm RCT conducted from March 2016 to May 2018 among ART clinic patients with hazardous alcohol use in Thai Nguyen – a mountainous, multi‐ethnic province located in Northeast Vietnam [49]. Mirroring Vietnam’s broader epidemic, HIV transmission in Thai Nguyen is primarily driven by injection drug use, with an HIV prevalence among people who inject drugs of 31.2% [50].

**Figure 1:**
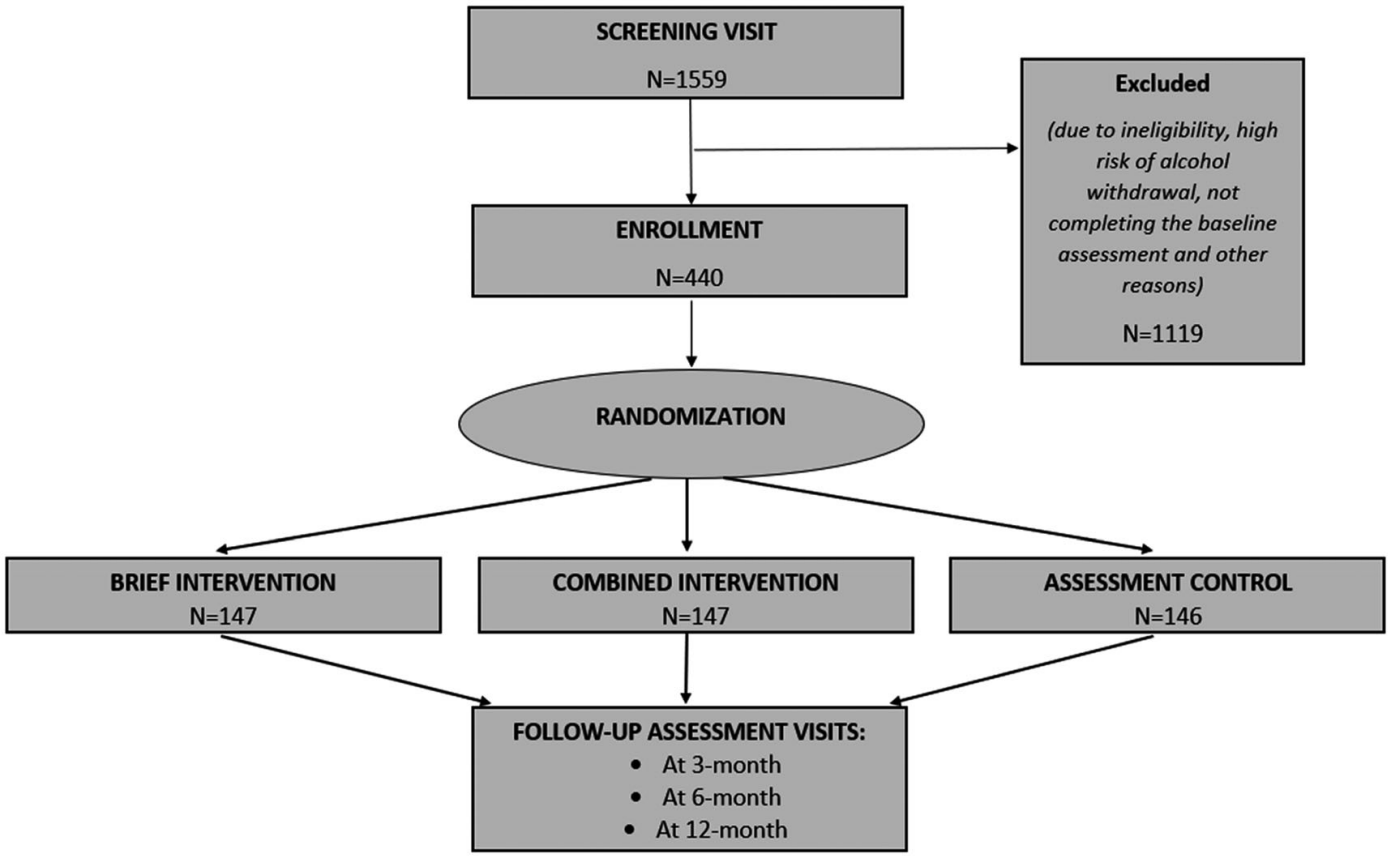
Study design of REDART intervention (Reducing Hazardous Alcohol Use & HIV Viral Load).

The main goal of the parent study was to understand the relative effectiveness of two interventions based on Motivational Enhancement Therapy (MET) and Cognitive Behavioral Therapy (CBT) in improving alcohol‐ and HIV‐related outcomes [49]. Four hundred and forty PWH with hazardous alcohol use were randomly assigned to receive either a combined intervention, a brief intervention or a standard of care assessment control (Figure 1). Participants were recruited from six ART community clinics and 1 ART hospital clinic. The World Health Organization (WHO) Alcohol Use Disorders Identification Test‐Concise (AUDIT‐C) survey, which had been utilized in previous studies in Vietnam [46, 51, 52, 53, 54], was used to assess eligibility [55]. Men and women who scored ≥4 (men) or ≥3 (women) on the AUDIT‐C were considered eligible [55]. Additional inclusion criteria were as follows: (1) being a current ART client; (2) being ≥18 years of age and (3) planning to reside in Thai Nguyen for the next 24 months. Exclusion criteria were as follows: (1) inability to provide informed consent due to cognitive impairment or having threatening behaviour (study staff assessed sobriety); (2) unwilling to provide locator information or (3) currently participating in other HIV, drug use or alcohol programme, study or intervention. Survey data, along with viral load data were collected at baseline, three, six and twelve months after the intervention. All questionnaires were administered in Vietnamese. The study was reviewed and approved by the University of North Carolina at Chapel Hill’s Institutional Review Board (IRB) and the IRB at the Thai Nguyen Center for Preventive Medicine.

The combined and brief interventions were associated with a significant improvement of the primary outcome – percent days abstinent, compared to the standard of care group at 12 months. VS (<20 copies of HIV‐1 RNA per millilitre) at 12 months was also higher after the brief intervention than the standard of care. Details of the main trial were published elsewhere [49].

### 2.2 Measurements

#### 2.2.1 Depression and anxiety symptoms

At all visits, depression symptoms were assessed with the Patient Health Questionnaire‐9 (PHQ‐9) scale [56] and anxiety symptoms were assessed with the Generalized Anxiety Disorder‐7 (GAD‐7) scale [57, 58]. In Vietnam, the PHQ‐9 scale has shown good convergent validity, construct validity as well as reliability [59], and has been used for a range of populations, including PWH [60, 61, 62]. Nine items assess how often depression symptoms including loss of interest or pleasure in doing things or feeling down or depressed occurred in the last two weeks. The GAD‐7 scale has not been formally validated in Vietnam but has been used in different Vietnamese populations to measure anxiety [63, 64]. This scale has seven items that evaluate the frequency of symptoms such as feeling nervous, anxious, or on edge or not being able to stop or control worrying. For both the PHQ‐9 and GAD‐7, a cut‐off score of 5 can be interpreted as having mild levels of symptoms that are consistent with depression and anxiety respectively [56, 65]. The maximum scores for depression and anxiety symptoms were 27 and 21 respectively. Since classifying continuous data into binary data can result in a loss of power and binary data are less sensitive to change [66], the original continuous scores of depression and anxiety symptoms were used in this study. Depression and anxiety symptoms were rescaled so that the reported estimates of association reflect the change in outcome associated with a 5‐unit change in the continuously measured PHQ‐9 or GAD‐7 score. We performed this rescaling because a 1‐unit change in each score is not clinically meaningful, whereas a 5‐unit change is considered potentially clinically significant, implying that a participant has moved from one level of severity to the next [67, 68]. This rescaling method has been used in other studies using continuous measures of depression and anxiety symptoms [69, 70].

#### 2.2.2 Alcohol dependence

Alcohol dependence was evaluated with the Mini International Neuropsychiatric Interview (MINI) questionnaire [71] – a 7‐item structured diagnostic psychiatric interview in which endorsing three or more items indicates alcohol dependence [72].

#### 2.2.3 VS and ART adherence

Viral load was measured by HIV‐1 ribonucleic acid (RNA) levels using the in vitro nucleic acid amplification test (COBAS^®^ AmpliPrep/COBAS^®^ TaqMan^®^ HIV‐1 Test). VS was defined as having less than 20 copies/ml. Complete ART adherence (self‐reported) was defined as no missed pills in the past three months. Both HIV viral load and self‐reported adherence were measured at baseline, three‐, six‐ and twelve‐month follow‐ups. Since ART adherence is subject to social desirability bias [73], VS was considered the primary outcome of interest in this study.

#### 2.2.4 Demographics and other covariates

Standard demographics were collected at baseline (e.g. age, marital status, education, employment). Based on the literature, the following covariates were chosen a priori as potential confounders: age, sex, education, marital status, employment, alcohol dependence, non‐injection drug use, injection drug use, social support, HIV stigma and intimate partner violence [16, 74, 75, 76, 77, 78, 79, 80, 81]. Participants were asked if they had used any types of non‐injection drugs (including heroin, methamphetamines, etc.) in the last three months and if they had ever injected drugs in the past. Social support was measured with a 5‐question modified version of the Medical Outcomes Study Social Support Instrument [82] used previously by our research group among PWH in Vietnam [83]. Based on the distribution of the social support score at baseline, the social support level was classified into four quartiles. To evaluate HIV stigma, participants were asked to state their levels of agreement with four statements indicating internalized, experienced or anticipated HIV stigma. They were classified as having HIV stigma if they reported any level of agreement with any of the four statements. Participants were classified as having ever experienced intimate partner violence if they had ever been a victim of physical, emotional or sexual abuse in an intimate relationship.

### 2.3 Statistical analysis

Means (standard deviations [SD]) of continuous variables and proportions of categorical variables were reported. Generalized estimating equations (GEE) models were used to estimate the time‐lagged associations between depression, anxiety symptoms and two HIV outcomes: VS and complete ART adherence. We conducted lagged analyses of the association between mental health symptoms at a given visit and HIV outcomes at the following visit. In this paper, time refers to the time of outcome assessment, which included: three‐month, six‐month or twelve‐month follow‐up visits. We estimated risk ratios (RR) of VS and ART adherence associated with a 5‐unit change in scores of depression and anxiety symptoms, as other studies have [69, 70]. Since depression and anxiety symptoms were highly correlated in our sample, for each outcome, separate models with the same set of covariates were run for depression or anxiety symptoms as the main predictor. Since HIV outcomes in the sample were very common (Table 1), the associations were explored using Poisson regression with robust variance estimation to avoid biases associated with inflated odds ratios [84]. Exchangeable covariance matrix between repeated measures was selected because it did not have convergence issues and had the smallest Quasi‐likelihood Information Criterion (QIC) [85].

**Table 1:** Participants' baseline characteristics, stratified by viral suppression and ART adherence at baseline

Characteristics N (%)	Complete ART adherence at baseline		Viral suppression at baseline		Total (N = 440)
	Yes (N = 334)	No (N = 103)	Yes (N = 370)	No (N = 70)	
Age (years) (mean ± SD)	40.8 ± 5.6	38.2 ± 5.8	40.3 ± 5.6	39.9 ± 6.6	40.2 ± 5.8
Male	322 (96.4)	101 (98.1)	361 (97.6)	65 (92.9)	426 (96.8)
Education					
*Primary school or less*	51 (15.3)	14 (13.6)	55 (14.9)	11 (15.7)	66 (15.0)
*Some secondary school*	191 (57.2)	53 (51.5)	201 (54.3)	45 (64.3)	246 (55.9)
*Some high school*	66 (19.8)	20 (19.4)	76 (20.5)	10 (14.3)	86 (19.6)
*Some technical training, college or university*	26 (7.8)	16 (15.5)	38 (10.3)	4 (5.7)	42 (9.6)
Marital status					
*Not married*	54 (16.2)	25 (24.3)	67 (18.1)	12 (17.1)	79 (18.0)
*Married*	245 (73.4)	57 (55.3)	261 (70.5)	44 (62.9)	305 (69.3)
*Widowed, divorced or separated*	35 (10.5)	21 (20.4)	42 (11.4)	14 (20.0)	56 (12.7)
Employment (Yes)	273 (81.2)	81 (78.6)	297 (80.3)	60 (85.7)	357 (81.1)
History of injection drug use (Yes)	267 (80.0)	87 (84.5)	301 (81.4)	55 (78.6)	356 (80.9)
Non‐injection drug use in the past three months (Yes)	125 (37.4)	46 (44.7)	145 (39.2)	27 (38.6)	172 (39.1)
Alcohol dependence (Yes)	55 (16.5)	36 (35.0)	82 (22.2)	11 (15.7)	93 (21.1)
Ever experienced, internalized or anticipated HIV stigma (Yes)	200 (59.9)	68 (66.0)	230 (62.2)	40 (57.1)	270 (61.4)
Ever experienced intimate partner violence (Yes)^a^	111 (33.5)	51 (50.5)	140 (38.3)	23 (33.3)	163 (37.5)
Social support (mean ± SD)	64.6 ± 28.9	61.1 ± 29.6	64.4 ± 29.2	60.7 ± 28.6	63.3 ± 29.27
Depression symptoms (mean ± SD)	2.6 ± 3.5	3.8 ± 4.2	2.9 ± 3.7	2.9 ± 3.9	2.9 ± 3.70
Anxiety symptoms (mean ± SD)	1.3 ± 2.5	2.4 ± 3.6	1.5 ± 2.8	1.9 ± 3.1	1.6 ± 2.85

ART, antiretroviral therapy; SD, standard deviation.

^a^ Five participants had missing data on intimate partner violence at baseline.

We assessed whether alcohol use modified the associations between mental health symptoms and HIV outcomes by adding interaction terms (e.g. depression symptoms × alcohol dependence) to the model. Similarly, the interactions between assessment time point and mental health symptoms were tested (e.g. depression symptoms × three‐month visit). Since the longitudinal effects of mental health symptoms on VS may vary by baseline VS, baseline VS was also examined as a potential effect modifier. Interactions with product terms not significantly different from 0 (at *p* < 0.05 using the Wald test) were not included in the multivariable regression models. Significant modification effects were further explored by probing the associations of interest within the stratum of the effect modifiers (controlling for confounders). Only covariates associated with the outcome at *p* < 0.1 in the univariable models and meaningfully changed the main estimates of association (by more than 10%) were included in the final models [86, 87]. Intervention exposure and time were kept in multivariable models regardless of statistical significance and meaningful change of the main estimates. Multiple imputations were used to accommodate missingness of depression, anxiety symptoms, VS and adherence data at follow‐ups [88, 89].

Statistical analyses were conducted using SAS 9.4 (SAS Institute, Inc., Cary, NC, USA).

## 3 Results

### 3.1 Sample characteristics at baseline

The mean age of enrolled participants (n = 440) was 40.2 years old (SD = 5.8) (Table 1). Almost all participants were male (96.8%), and 85% had at least some secondary school education. More than two‐thirds (69.3%) were married, and 81.1% were employed at baseline. Most participants had a history of injection drug use (80.9%), and 39.1% had used non‐injection drugs in the past three months. Alcohol dependence based on the MINI score was identified among 21.1% of participants. More than half had ever experienced HIV stigma (61.4%), and 37.5% had ever experienced intimate partner violence. The mean social support score was 63.3 (SD = 29.3, scale 0 to 100).

At baseline, 84.1% were virally suppressed and 76.4% had not missed an ART pill in the last three months. There was no difference in depression symptoms between participants with and without VS at baseline. A slightly lower score for anxiety symptoms was observed among those with VS (Table 1). Those who completely adhered to their ART regimen at baseline had lower depression and anxiety symptoms scores, compared to those without complete adherence.

### 3.2 Distribution of depression, anxiety symptoms and HIV outcomes over time

**Figure 2:**
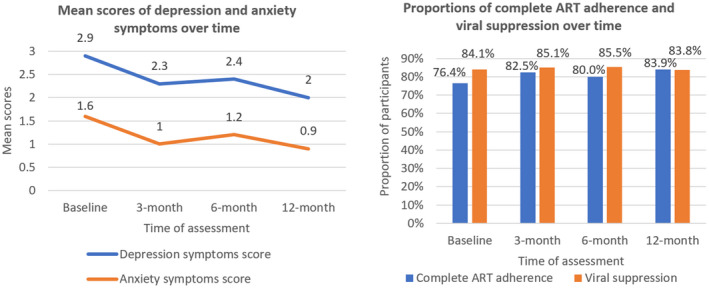
Changes in depression, anxiety symptoms, viral suppression and complete ART adherence of the sample over time. ART, antiretroviral therapy.

Figure 2 shows changes in mental health symptoms, VS and ART adherence of the whole sample over time. There is a decrease in observed depression and anxiety symptoms from baseline to 12‐month follow‐up. There were no significant changes in VS over time, whereas complete ART adherence increased from 76.4% at baseline to 84.8% at the last follow‐up.

### 3.3 Associations between depression, anxiety symptoms and HIV outcomes

Overall, there were no associations between mental health symptoms and VS. However, a 5‐point increase in depression or anxiety symptoms score was associated with a lower probability of complete ART adherence at the next visit (depression symptoms: adjusted risk ratio (aRR) = 0.95; 95% CI: 0.91 to 0.99; anxiety symptoms: aRR = 0.93; 85% CI: 0.88 to 0.99) (Table 2).

**Table 2:** Associations between depression, anxiety symptoms and HIV outcomes at the next visit^a^

	Viral suppression^b^			ART adherence^c^		
	aRR	95%CI	*p*‐values	aRR	95%CI	*p*‐values
Depression symptoms	1.00	0.96 to 1.03	0.94	0.95	0.91 to 0.99	0.03
Anxiety symptoms	1.00	0.95 to 1.05	0.98	0.93	0.88 to 0.99	0.02

ART, antiretroviral therapy; aRR, adjusted risk ratio; CI, confidence interval.

^a^ Each multivariable model has only one mental health predictor, either depression symptoms or anxiety symptoms; models with the same outcome have the same set of covariates. aRRs were associated with a 5‐point increase in scores of depression or anxiety symptoms at the previous time point

^b^ models predicting viral suppression controlled for age, viral suppression at baseline, intervention exposure and time

^c^ models predicting adherence controlled for marital status, alcohol dependence at baseline, adherence at baseline, intervention exposure and time

There was a significant effect modification by time, and the strength and significance of the associations between anxiety symptoms and HIV outcomes varied across study time points. Estimates of the full models with *p*‐values of interaction terms are presented in Supplementary File, Table S1. When being stratified by time, both baseline depression and anxiety symptoms were associated with a lower probability of complete ART adherence at three months (depression symptoms: aRR = 0.90; 95%CI: 0.84 to 0.97; anxiety symptoms: aRR = 0.87; 85%CI: 0.79 to 0.96), though no association was seen with VS. At subsequent follow‐up visits, there was no association between symptoms of depression and anxiety with either VS or complete ART adherence, except for a signal of higher VS at 12 months associated with a 5‐point increase in anxiety symptoms at six months (aRR = 1.09; 95%CI 1.02 to 1.17) (Table 3). Baseline VS was not a significant modifier of the associations between depression, anxiety and VS (Tables S2.1 and S2.2).

**Table 3:** Associations between depression, anxiety symptoms and HIV outcomes at the next visit, stratified by time of outcome assessment^a^

Time of outcome assessment	Models predicting viral suppression^b^			Models predicting complete ART adherence^c^		
	aRR	95%CI	*p*‐values	aRR	95%CI	*p*‐values
Main predictor: depression symptoms						
At three‐month visit	0.99	0.94 to 1.04	0.66	0.90	0.84 to 0.97	0.005
At six‐month visit	0.97	0.91 to 1.03	0.31	0.96	0.89 to 1.03	0.28
At twelve‐month visit	1.05	1.00 to 1.10	0.09	1.01	0.94 to 1.09	0.69
Main predictor: anxiety symptoms						
At three‐month visit	0.96	0.89 to 1.03	0.30	0.87	0.79 to 0.96	0.005
At six‐month visit	0.96	0.86 to 1.07	0.50	0.90	0.79 to 1.03	0.14
At twelve‐month visit	1.09	1.02 to 1.17	0.02	1.04	0.96 to 1.13	0.36

ART, antiretroviral therapy; aRR, adjusted risk ratio; CI, confidence interval.

^a^ Each multivariable model has only one mental health predictor, either depression symptoms or anxiety symptoms at the previous time point. Models with the same outcome have the same set of covariates. Time presented in the first column is the time of assessment of viral suppression and ART adherence. aRRs were associated with a 5‐point increase in scores of depression or anxiety symptoms at the previous time point

^b^ models predicting viral suppression control for age, viral suppression at baseline, intervention exposure, time, interaction of time × depression/anxiety symptoms

^c^ models predicting adherence control for marital status, alcohol dependence at baseline, adherence at baseline, intervention exposure, time, interaction of time × depression/anxiety symptoms

### 3.4 Associations between depression/anxiety symptoms and HIV outcomes: Possible modification role of alcohol dependence

Alcohol dependence at baseline significantly modified the association between anxiety symptoms and complete ART adherence (*p*‐value for interaction term = 0.02). Anxiety symptoms at baseline and lower probability of complete adherence at three months were more strongly related among participants with alcohol dependence, compared to those without (Table 4). Anxiety symptoms only predicted poor adherence at six months among those with alcohol dependence. At 12 months, there was no association between anxiety at the previous time point and adherence for all participants. The interactions between alcohol dependence and mental health symptoms in the remaining associations (depression predicting both HIV outcomes and anxiety predicting VS) were not significant at *p* < 0.05 and were not further explored.

**Table 4:** Associations between anxiety symptoms and complete ART adherence at the next visit, stratified by alcohol dependence and time since baseline

Time of outcome assessment	aRR^a^	95%CI	*p*‐values
At three‐month visit			
Alcohol dependence	0.80	0.68 to 0.94	0.008
No alcohol dependence	0.90	0.82 to 0.99	0.04
At six‐month visit			
Alcohol dependence	0.82	0.68 to 0.99	0.05
No alcohol dependence	0.93	0.82 to 1.06	0.33
At twelve‐month visit			
Alcohol dependence	0.95	0.81 to 1.12	0.57
No alcohol dependence	1.08	1.00 to 1.17	0.08

aRR, adjusted risk ratio; CI, confidence interval

^a^ Models controlling for marital status, alcohol dependence at baseline, adherence at baseline, intervention exposure, time, interaction of time × anxiety symptoms, interaction of alcohol dependence × anxiety symptoms. aRRs were associated with 5‐point increase in scores of anxiety symptoms at the previous time point.

## 4 Discussion

Among a sample of 440 ART clients with hazardous alcohol use, we did not find an overall effect of depression and anxiety symptoms on VS, but observed a decreased probability of complete ART adherence associated with increased depression and anxiety symptoms. The magnitude and significance of the associations varied by the time of outcome assessment. Negative effects of anxiety symptoms on ART adherence were significantly worse among participants with alcohol dependence at baseline, compared to those without alcohol dependence.

While there was no effect of mental health symptoms on VS when all time points were taken into account, we found that anxiety symptoms were associated with a small increase in the probability of VS at the last follow‐up. This was an unexpected finding, although one study among PWH in Russia also reported that a greater state of anxiety was positively associated with better adherence [16]. Previous studies reported a Hawthorne effect, which refers to the alteration of behaviour of subjects due to awareness of being observed in a study [90, 91]. Moreover, an inverted U‐shaped association between arousal, anxiety and performance [92, 93] has been demonstrated. By participating in multiple rounds of interviews, participants with milder anxiety symptoms might become more aware of and worried about their health’s status, and therefore more motivated to take action to improve their overall health. Participants with more severe anxiety were more likely to be lost to follow‐up in this study (data not shown) – therefore at 12‐month follow‐up, participants with lower levels of anxiety might comprise a greater proportion of the sample.

Our results are similar to other studies that examined the association between mental health symptoms and ART adherence in the general population of PWH [17, 19]. However, the magnitudes of the associations in our study were smaller than those reported by previous studies. Participants in our sample had a high proportion of VS and ART adherence at baseline, which leaves fewer opportunities for enhancement of HIV outcomes. Due to the Hawthorne effect mentioned above, being a participant in REDART over time might have also attenuated the effects of mental health problems on adherence and viral suppression to some extent, regardless of which intervention arm the participant belonged to. This might help explain why stratification by time only showed significant associations between mental health symptoms and ART adherence at the three‐month visit.

We also found that the negative effects of anxiety symptoms on ART adherence at earlier time points were modified by alcohol dependence such that the associations appeared to be stronger among those with alcohol dependence. Previous studies among PWH reported that a higher number of syndemic conditions was associated with higher HIV viral load and lower ART adherence [94, 95], although the authors did not examine the specific interaction of alcohol use and mental health symptoms. PWH can use alcohol as a coping strategy, which may help improve mood to an extent [44]. However, since hazardous alcohol use independently decreased ART adherence [96, 97], high levels of alcohol use such as alcohol dependence among PWH with anxiety symptoms may pose greater challenges than benefits to adherence.

Our analyses have several limitations. First, self‐reported measures of adherence are more likely to produce measurement errors, as compared to objective measures such as electronic medication packaging devices [73]. In order to minimize this limitation, we also analysed the associations between mental health symptoms and HIV VS – a biological outcome not subject to such biases. Second, our study was not immune to loss to follow‐up – a common issue affecting longitudinal analyses. We had missing data for key predictors and outcomes at the follow‐up visits, ranging from 7% to 12% (Table S3). In this study, we used multiple imputations to impute missing values of depression, anxiety symptoms, VS and adherence for the sample, as recommended for GEE analyses of longitudinal data [88, 89]. Third, our sample was not a random sample of ART clients. The majority of our participants were men and had a history of injection drug use. HIV transmission in Thai Nguyen is primarily driven by injection drug use [50] – a behaviour more commonly seen among men in Vietnam [98, 99]. Other studies among PWH in Vietnam also reported overwhelming proportions of participants being male with drug use behaviours [46, 53]. Finally, in this study, we reported aRRs associated with a 5‐unit change in depression and anxiety symptoms. We acknowledge that there are alternative ways to analyse PHQ‐9 and GAD‐7 scores, which might result in different estimates of the associations between mental health symptoms and HIV outcomes than ours.

Our findings suggest that increased depression or anxiety symptoms over time are associated with decreased ART adherence among PWH with hazardous alcohol use, and support a modifying effect of alcohol dependence on the association between anxiety symptoms and ART adherence in this group. We recommend that future interventions aim to raise awareness about mental health problems among PWH, especially those with alcohol use disorders. Mental health services such as screening, counselling or medication treatment are also imperative to improve HIV outcomes for PWH with hazardous alcohol use. These mental health services can be integrated into alcohol use interventions or into existing HIV primary care clinics in Vietnam. It is also important that these interventions are tested for efficacy and cost‐effectiveness in low‐resource settings such as Vietnam.

## 5 Conclusions

Depression and anxiety symptoms had no overall effect on VS, although anxiety symptoms at six months were associated with a mild increase in the probability of VS at 12 months. Increased depression and anxiety symptoms were associated with a lower probability of complete ART adherence, and participants with both alcohol dependence and anxiety symptoms had the lowest adherence. Interventions focusing on mental healthcare for PWH with hazardous alcohol use are much needed, and optimal models integrating mental healthcare and alcohol reduction should be implemented and tested in HIV primary care clinics in low‐resource settings.

## Competing interest

The authors have declared no conflict of interest.

## Authors’ contributions

V.G., C.L., H.H., G.C., K.L., T.S., H.T. and D.D. performed the research. V.G., C.L., H.H., G.C., K.L., D.D. and C.F. designed the research study. M.N. analysed the data and wrote the paper. V.G., H.L.R., B.P. K.M., C.L., D.D., H.H., G.C. and K.L. revised it critically for important intellectual contents. All authors have given final approval of the manuscript to be published.

## Supporting information


**Figure S1.** Conceptual model and mapping of underlying theories
**Table S1.** Associations between depression, anxiety symptoms and HIV outcomes at the next visit, taking into account the effect modification by time (Models with interaction terms)
**Table S2.** Effect modification of baseline viral suppression on the associations between depression, anxiety symptoms and viral suppression
**Table S3.** Missing data of depression, anxiety symptoms and HIV outcomes at follow‐up visits
