# Supplementary material for: The longitudinal association between depression, anxiety symptoms and HIV outcomes, and the modifying effect of alcohol dependence among ART clients with hazardous alcohol use in Vietnam
Source: J Int AIDS Soc. 2021 Jun 24;24(Suppl 2):e25746. doi: 10.1002/jia2.25746 (PMC8222856; doi:10.1002/jia2.25746)
Supplement: Supplementary file 1 — Figure S1. Conceptual model and mapping of underlying theories Table S1. Associations between depression, anxiety symptoms and HIV outcomes at the next visit, taking into account the effect modification by time (Models with interaction terms) Table S2. Effect modification of baseline viral suppression on the associations between depression, anxiety symptoms and viral suppression Table S3. Missing data of depression, anxiety symptoms and HIV outcomes at follow‐up visits [file JIA2-24-e25746-s001.docx]

# Supplementary File

## Figure S1: Conceptual model and mapping of underlying theories


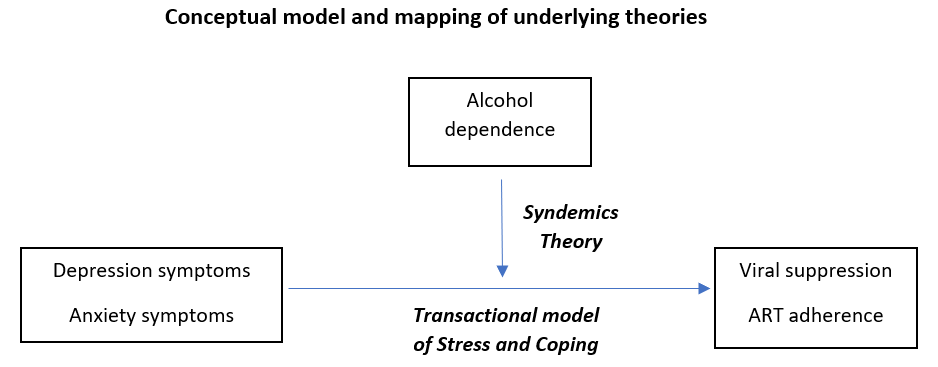


## Table S1: Associations between depression, anxiety symptoms and HIV outcomes at the next visit, taking into account the effect modification by time (Models with interaction terms)

| **Table S1. Associations between depression, anxiety symptoms and HIV outcomes at the next visit¹** | | | | | | | |
| --- | --- | --- | --- | --- | --- | --- | --- |
|  | **Viral supression²** | | | **ART adherence³** | | |  |
|  | **aRR** | **95%CI** | **p-values** | **aRR** | **95%CI** | **p-values** |  |
| **Main predictor: Depression symptoms** | | | | | | |  |
| Depression symptoms (at the previous timepoint) | 1.07 | 0.77-1.47 | 0.7 | 0.90 | 0.85-0.96 | 0.002 |  |
| Time (of outcome assessment) |  |  |  |  |  |  |  |
| *3-month* | 1.00 | *** | *** | 1.00 | *** | *** |  |
| *6-month* | 1.01 | 0.95-1.08 | 0.7 | 0.93 | 0.87-0.99 | 0.04 |  |
| *12-month* | 0.96 | 0.90-1.02 | 0.19 | 0.96 | 0.89-1.02 | 0.19 |  |
| Interaction terms |  |  |  |  |  |  |  |
| *Depression symptoms x 6-month* | 0.98 | 0.90-1.06 | 0.58 | 1.06 | 0.97-1.16 | 0.22 |  |
| *Depression symptoms x 12-month* | 1.06 | 0.98-1.15 | 0.16 | 1.12 | 1.03-1.23 | 0.01 |  |
| *Wald test for joint significance*⁴ |  |  | 0.13 |  |  | 0.05 |  |
| **Main predictor: Anxiety symptoms** | | | | | | |  |
| Anxiety symptoms (at the previous timepoint) | 0.96 | 0.89-1.04 | 0.29 | 0.87 | 0.80-0.95 | 0.002 |  |
| Time (of outcome assessment) |  |  |  |  |  |  |  |
| *3-month* | 1.00 | *** | *** | 1.00 | *** | *** |  |
| *6-month* | 1.00 | 0.94-1.06 | 0.97 | 0.95 | 0.89-1.01 | 0.09 |  |
| *12-month* | 0.95 | 0.90-1.01 | 0.1 | 0.97 | 0.91-1.03 | 0.28 |  |
| Interaction terms |  |  |  |  |  |  |  |
| *Anxiety symptoms x 6-month* | 1.00 | 0.89-1.13 | 0.95 | 1.03 | 0.90-1.18 | 0.63 |  |
| *Anxiety symptoms x 12-month* | 1.14 | 1.02-1.27 | 0.02 | 1.20 | 1.06-1.36 | 0.003 |  |
| *Wald test for joint significance*⁴ |  |  | 0.04 |  |  | 0.01 |  |
| *¹Each multivariable model has only one mental health predictor, either depression symptoms or anxiety symptoms; models with the same outcome have the same set of covariates. aRRs were associated with a 5-point increase in scores of depression or anxiety symptoms at the previous time point ²Models predicting viral suppression controlled for age, viral suppression at baseline, intervention exposure, time, interaction of time*depression/anxiety symptoms ³Models predicting adherence controlled for marital status, alcohol dependence at baseline, adherence at baseline, intervention exposure, time, interaction of time*depression/anxiety symptoms* ⁴*p-value of the Wald test for joint significance of all interaction terms in the model Note: ART: antiretroviral therapy; aRR: adjusted risk ratio; CI: confidence interval* | | | | | | |  |

## Table S2: Effect modification of baseline viral suppression on the associations between depression, anxiety symptoms and viral suppression

| **Table S2.1. Effect modification of baseline viral suppression on the associations between depression, anxiety symptoms and viral suppression¹** | | | |
| --- | --- | --- | --- |
|  | **aRR** | **95%CI** | **p-values** |
| **Main predictor: Depression symptoms** | | | |
| *Depression symptoms* | 0.99 | 0.95-1.02 | 0.42 |
| *Depression symptoms x Viral suppression at baseline* | 0.89 | 0.79-0.99 | 0.04 |
| **Main predictor: Anxiety symptoms** | | | |
| *Anxiety symptoms* | 0.98 | 0.93-1.03 | 0.44 |
| *Anxiety symptoms x Viral suppression at baseline* | 0.88 | 0.76-1.01 | 0.07 |
| *¹All models controlling for age, viral suppression at baseline, intervention exposure, time, interaction of baseline viral suppression*depression/anxiety symptoms Note: aRR: adjusted risk ratio; CI: confidence interval* | | | |

| **Table S2.2. Associations between depression symptoms and viral suppression, stratified by baseline viral suppression¹** | | | |
| --- | --- | --- | --- |
|  | **aRR** | **95%CI** | **p-values** |
| *Viral suppression at baseline* | 0.99 | 0.96-1.01 | 0.32 |
| *No viral suppression at baseline* | 1.11 | 0.98-1.27 | 0.14 |
| *¹All models controlling for age, viral suppression at baseline, intervention exposure, time, interaction of baseline viral suppression*depression/anxiety symptoms Note: aRR: adjusted risk ratio; CI: confidence interval* | | | |

## Table S3: Missing data of depression, anxiety symptoms and HIV outcomes at follow-up visits

| **Table S3. Missing data of depression, anxiety symptoms and HIV outcomes at follow-up visits** | | | | | | |
| --- | --- | --- | --- | --- | --- | --- |
|  | ***3-month*** | | ***6-month*** | | ***12-month*** | |
|  | ***N*** | ***%*** | ***N*** | ***%*** | ***N*** | ***%*** |
| **Mental health** |  |  |  |  |  |  |
| *Depression symptoms* | 35 | 8 | 30 | 7 | 50 | 11 |
| *Anxiety symptoms* |  |  |  |  |  |  |
| **HIV outcomes** |  |  |  |  |  |  |
| *Complete ART adherence* | 40 | 9 | 39 | 8 | 53 | 12 |
| *Viral suppression* | 37 | 8 | 30 | 7 | 50 | 11 |

*Note: ART: antiretroviral therapy*
